# Determination of Histidine Protonation States in Proteins by Fast Magic Angle Spinning NMR

**DOI:** 10.3389/fmolb.2021.767040

**Published:** 2021-12-10

**Authors:** Roman Zadorozhnyi, Sucharita Sarkar, Caitlin M. Quinn, Kaneil K. Zadrozny, Barbie K. Ganser-Pornillos, Owen Pornillos, Angela M. Gronenborn, Tatyana Polenova

**Affiliations:** ^1^ Department of Chemistry and Biochemistry, University of Delaware, Newark, DE, United States; ^2^ Pittsburgh Center for HIV Protein Interactions, University of Pittsburgh School of Medicine, Pittsburgh, PA, United States; ^3^ Department of Molecular Physiology and Biological Physics, University of Virginia School of Medicine, Charlottesville, VA, United States; ^4^ Department of Structural Biology, University of Pittsburgh School of Medicine, Pittsburgh, PA, United States

**Keywords:** Magic angle spinning (MAS), nuclear magnetic resonance (NMR) spectroscopy, histidine protonation state, transferred echo double resonance (TEDOR), Fast MAS NMR, solid-state NMR

## Abstract

Histidine residues play important structural and functional roles in proteins, such as serving as metal-binding ligands, mediating enzyme catalysis, and modulating proton channel activity. Many of these activities are modulated by the ionization state of the imidazole ring. Here we present a fast MAS NMR approach for the determination of protonation and tautomeric states of His at frequencies of 40–62 kHz. The experiments combine ^1^H detection with selective magnetization inversion techniques and transferred echo double resonance (TEDOR)–based filters, in 2D heteronuclear correlation experiments. We illustrate this approach using microcrystalline assemblies of HIV-1 CA_CTD_-SP1 protein.

## Introduction

**FIGURE 1:**
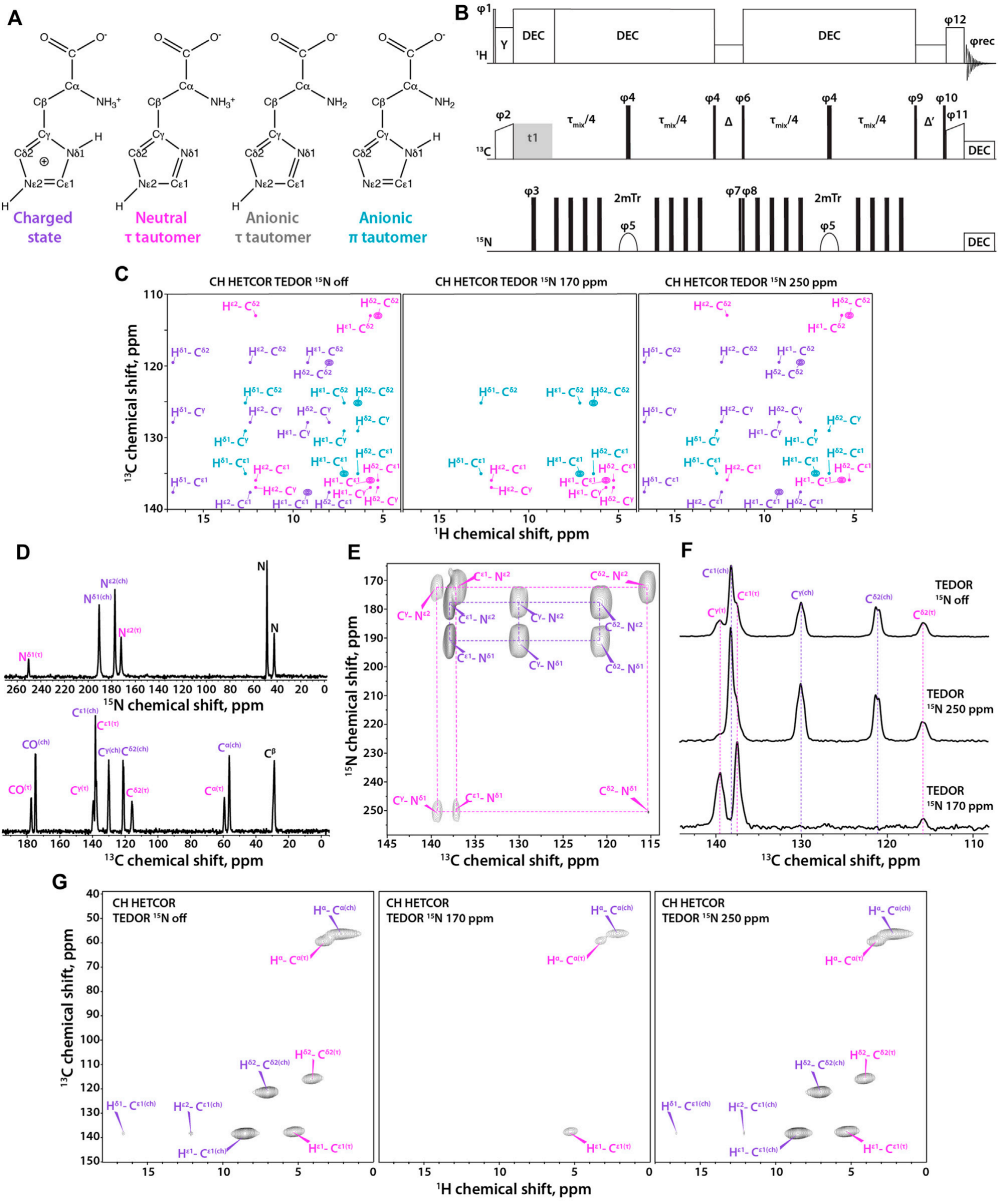
**(A)** Four states of histidine: left to right, charged state, neutral τ tautomer, anionic τ tautomer, and anionic π tautomer. **(B)** Pulse sequence for the ^1^H-detected TEDOR-based ^15^N selective filtered experiment. T_r_ is the MAS rotor period, τ_mix_ is the total TEDOR mixing time. The phase on the individual pulses are: φ1 = 16 × (0) 16 × (2), φ2 = 1, φ3 = 0,φ4 = 0, φ5 = 0213 2031, φ6 = 2, φ7 = 0, φ8 = 02, φ9 = 1133, φ10 = 4 × (0) 4 × (1) 4 × (2) 4 × (3), φ11 = 4 × (1) 4 × (0), φ12 = 4 × (1) 4 × (0) 4 × (1) 4 × (0) 4 × (3) 4 × (2) 4 × (3) 4 × (2), φrec = 3113 0220 1331 2002, where 0 = *x*, 1 = *y*, 2 = –*x*, and 3 = –*y*. Δ is set to one rotor period during which ^1^H rf field of ω_r_ amplitude is applied for effective Z-filtering. MISSISSIPPI water suppression sequence is applied during Δ′ time period. **(C)** Synthetic ^1^H-detected TEDOR-based ^15^N selective filtered CH HETCOR spectra showing cross peaks expected for each tautomer. Left to right, soft pulse turned off, soft pulse at 170 ppm, soft pulse at 250 ppm. The filtering patterns for neutral and anionic τ tautomers are identical. **(D)** ^15^N **(top)** and ^13^C **(bottom)** CPMAS NMR spectra of crystalline histidine. **(E)** Aromatic region expansion of 2D NCA spectrum of crystalline histidine. **(F)** 1D ^13^C spectra using TEDOR-based ^15^N selective filtering in the aromatic region. Top to bottom, soft pulse turned off; soft pulse at 250 ppm; soft pulse at 170 ppm. **(G)** Three complementary ^1^H-detected TEDOR-based ^15^N selective filtered CH HETCOR spectra. Left to right, soft pulse turned off, soft pulse at 170 ppm, soft pulse at 250 ppm. The MAS frequency was 60 kHz in all experiments. Signals of charged state are shown in purple, neutral τ tautomer – in magenta, anionic τ tautomer - in grey, and anionic π tautomer – in teal.

Histidines (His) play important structural and functional roles in proteins such as metal binding (Stryer et al., 1964; Perutz and Mathews, 1966; Adams et al., 1969; Liljas et al., 1972), proton transfer (Hoffee et al., 1967; Blow et al., 1969; Campbell et al., 1974), and stability (Perutz et al., 1969; Lewis et al., 1976; Loewenthal et al., 1992). These functions are often correlated with the ionization state of the histidine sidechain (Figure 1A) (Bachovchin and Roberts, 1978; Kossiakoff and Spencer, 1981; Lewis et al., 1981). While the pK_a_ of the imidazole ring for free histidine is 6.5 (Blomberg et al., 1977), in proteins the pK_a_ values vary widely, from 3 to 9, depending on the interactions with neighboring residues and degree of burial (Zhou et al., 1993; Plesniak et al., 1996). At pH values above the pK_a_, anionic τ and π tautomers with hydrogens at either N^ε2^ or N^δ1^ are present, while below the pK_a_ the protonated imidazole ring possesses hydrogens at both N^ε2^ and N^δ1^. For a protein at intermediate pH values, it is possible that a fraction of His residues is protonated and the remaining fraction unprotonated (French and Hammes, 1965; Edwards and Sykes, 1980; Hass et al., 2008).

Methods to determine His ionization states in proteins are solution NMR (Kilmartin et al., 1973; Markley, 1975; Bachovchin and Roberts, 1978; Perutz et al., 1985; Pelton et al., 1993; Shimba et al., 1998; Hass et al., 2008; Hansen and Kay, 2014) or neutron diffraction (Kossiakoff and Spencer, 1980; Maeda et al., 2004; Kovalevsky et al., 2010), with the latter limited to very large single crystals and requiring a neutron source, both difficult conditions to meet routinely. Therefore, solid-state magic angle spinning (MAS) NMR constitutes a viable alternative (Wei et al., 1999). Similar to solution NMR, the tautomeric state of histidines can be unambiguously determined from a unique combination of ^15^N sidechain chemical shifts (Munowitz et al., 1982; Wei et al., 1999; Miao et al., 2014) and the corresponding N-H distances can be estimated, allowing for hydrogen bonding studies (Shenderovich et al., 2015). Protonation states for the crystalline histidine amino acid have been determined by MAS NMR for different pH values (Li and Hong, 2011) and crystalline short peptides (Platzer et al., 2014). Using ^15^N selective filtered, ^13^C-detected experiments with the inversion pulses at frequencies of the different tautomers (Miao et al., 2014) permits their identification. For proteins containing several histidine residues, the above experiments are challenging due to low sensitivity and spectral overlap. Therefore, only a handful of such studies have been reported to date (Hu et al., 2006; Hu et al., 2010; Miao et al., 2015; Kwon et al., 2019; Maciejko et al., 2019; Vasa et al., 2019; Movellan et al., 2020). In order to increase resolution, the original pulse sequence can be reconfigured as a 2D experiment by introducing a ^13^C-^13^C mixing period based on proton-driven spin diffusion (PDSD) (Bloembergen, 1949) and extending the second Z-filter (Miao et al., 2014). 2D and 3D proton-based experiments were also introduced with ^1^H chemical shifts either recorded in the indirect dimension (Miao et al., 2015) or detected directly (Shenderovich et al., 2015; Vasa et al., 2019; Movellan et al., 2020).

Herein, we present an alternative MAS experiment that uses ^1^H detected transferred-echo double resonance (TEDOR)-based ^15^N selectively filtered 2D correlations at fast MAS frequencies of 40–60 kHz. The advantages of the ^1^H-detected fast-MAS experiments presented here are: i) improved sensitivity due to ^1^H detection, and ii) improved resolution *via* the second dimension and selective recoupling of aromatic resonances directly attached to ^15^N atoms. Microcrystalline assemblies of U-^13^C,^15^N- and fractionally deuterated (FD) (Mance et al., 2015) ^13^C,^15^N-HIV-1 CA_CTD_-SP1 protein samples, possessing solely a single His residue, His-226, are ideally suited for pulse sequence optimization and therefore were selected for illustrating our current approach. Extension to ultrafast MAS frequencies (up to 110 kHz), should yield even higher sensitivity and resolution for proteins with multiple histidines.

## Materials and Methods

### Sample Preparation

U-^13^C,^15^N-L-histidine was purchased from Cambridge Isotope Laboratories, recrystallized from an aqueous solution at pH 6.0, adjusted by mixing HCl and NaOH. The sample was packed into a 1.3 mm MAS rotor. Microcrystalline assemblies of U-^13^C,^15^N- and FD-^13^C,^15^N-HIV-1 CA_CTD_-SP1 were prepared in the presence of the assembly cofactor inositol hexakisphosphate (IP6) as described previously (Wagner et al., 2016) except for growing *Escherichia coli* in M9 medium containing ^13^C glucose, ^15^N NH_4_Cl, isotopically labeled precursors, and (for the deuterated sample) D_2_O. Proteins were assembled with 1.6 mM IP6 (Sigma-Aldrich), for a final reaction volume of 1 ml at pH 8.0. Assemblies were incubated overnight at 20°C and packed into 3.2 mm (U-^13^C,^15^N), 1.9 mm (FD-^13^C,^15^N), or 1.3 mm MAS rotors (U-^13^C,^15^N).

### MAS NMR Spectroscopy

MAS NMR experiments on U-^13^C,^15^N-CA_CTD_-SP1 and FD-^13^C,^15^N-CA_CTD_-SP1 microcrystalline assemblies were performed on a 20.0 T Bruker AVIII spectrometer outfitted with 3.2 mm E-Free HCN and 1.9 HCN probes, respectively. The MAS frequency was 14 and 40 kHz, respectively, controlled to within ± 10 Hz by a Bruker MAS controller. The actual sample temperature was maintained at 4 ± 1°C throughout the experiments using the Bruker temperature controller.

The Larmor frequencies were 850.4 MHz (^1^H), 213.9 MHz (^13^C) and 86.2 MHz (^15^N). The typical 90° pulse lengths were 2.6–3.0 μs for ^1^H, 4.3–4.5 μs for ^13^C, and 4.2–4.7 μs for ^15^N. The ^1^H-^13^C and ^1^H-^15^N cross-polarization employed a linear amplitude ramp of 90–110% on ^1^H, and the center of the ramp was matched to a Hartmann–Hahn condition at the first spinning sideband; contact times of 0.7–1.5 ms and 1.0–1.7 ms were used, respectively. 50 ms CORD (Hou et al., 2013) mixing time was applied to facilitate ^13^C-^13^C mixing.

MAS NMR experiments on U-^13^C,^15^N-L-histidine and FD-^13^C,^15^N-CA_CTD_-SP1 microcrystalline assemblies were performed on a 14.1 T Bruker AVIII spectrometer outfitted with 1.3 mm HCN probe. Larmor frequencies were 599.8 MHz (^1^H), 150.8 MHz (^13^C), and 60.7 MHz (^15^N). The MAS frequency was 60 kHz, controlled to within ± 10 Hz by a Bruker MAS controller. The actual sample temperature was maintained at 40 ± 1°C throughout the experiments using the Bruker temperature controller. The typical 90° pulse lengths were 1.4–1.6 μs for ^1^H, 2.7–3.0 μs for ^13^C, and 3.3–3.6 μs for ^15^N. The ^1^H-^13^C and ^1^H-^15^N cross-polarization employed a linear amplitude ramp of 90–110% on ^1^H, center of the ramp was matched to a Hartmann–Hahn condition at the first spinning sideband, with contact times of 1.0–5.0 ms and 1.3–5.0 ms, respectively. Band-selective ^15^N-^13^C SPECIFIC-CP contact time was 5.0–6.0 ms. SWFTPPM (Vinod Chandran et al., 2008) decoupling (15 kHz) was used during the TEDOR block and acquisition periods. The selective ^15^N 180° r-SNOB (Kupce et al., 1995) pulse length in the Z-filtered TEDOR experiments was 500 µs and the bandwidth — 2 kHz; the rf power was 4 kHz. During the Z-filter time period Δ, 60 kHz CW decoupling was applied for τ_r_ on ^1^H channel, while during the time period Δ’, MISSISSIPPI (Zhou and Rienstra, 2008) water suppression was applied. The TEDOR block duration was 1–3 ms.

### Data Processing

All MAS NMR data were processed using NMRPipe (Delaglio et al., 1995). The ^13^C and ^15^N chemical shifts were referenced with respect to the external standards adamantane (Morcombe and Zilm, 2003) and ammonium chloride (Bertani et al., 2014), respectively. The 2D and 3D data sets were processed by applying 30, 45, 60, and 90° shifted sine bell apodization followed by a Lorentzian-to-Gaussian transformation in both dimensions. Forward linear prediction to twice the number of the original data points was used in the indirect dimension followed by zero filling. The processed spectra were analyzed in NMRFAM-Sparky (Goddard and Kneller, 2004; Lee et al., 2015) and CCPN (Stevens et al., 2011).

## Results

Here, we report on a 2D ^1^H-detected TEDOR-based Z-filtered experiment, which incorporates ^15^N selective filters for the determination of histidine tautomeric states. The pulse sequence is shown in Figure 1B. The experiment is well suited for fast MAS frequencies of 40 kHz and above. The tautomeric states of His residues are unambiguously determined using a combination of three CH HETCOR experiments comprising: i) ^15^N selective TEDOR filter, containing ^13^C resonances of all protonation and tautomeric states present; ii) ^15^N selective TEDOR filter with a soft pulse at 170 ppm, removing resonances of the protonated state while C^ε1^ and C^δ2^ atoms of π tautomer and C^ε1^ and C^γ^ atoms of τ tautomer remain; and iii) ^15^N selective TEDOR filter with a soft pulse at 250 ppm, retaining all signals of the charged state, C^ε1^ and C^γ^ of the π tautomer as well as C^ε1^ and C^δ2^ atoms of the τ tautomer. C^ε1^ of anionic tautomers is always present in TEDOR filtered spectra, but has reduced peak intensity when ^15^N selective pulse is applied as C-N dipolar interaction with the non-selectively irradiated nitrogen atom is recoupled. The sequence was first tested on a crystalline L-histidine sample prepared at pH 6.0. The ^13^C and ^15^N 1D CPMAS and 2D NCA spectra are shown in Figures 1D, E, respectively. The spectra clearly indicate the presence of two forms of L-histidine, the charged monohydrate and the τ tautomer, in approximately 2:1 ratio. As shown in Figure 1F, conventional ^13^C-detected TEDOR-based experiments are well suited for the determination of protonation states in this sample. To test the ^1^H-detected sequences proposed herein, three complementary experiments were performed. As shown in Figure 1G, ^15^N selective TEDOR-filtered CH HETCOR without or with a soft pulse at 250 ppm (left and right panels, respectively) yield the sidechain signals of both protonation states, while ^15^N selective TEDOR-filtered CH HETCOR with soft pulse at 170 ppm retains only C^ε1^ resonance of the τ tautomer (chemical shifts provided in Supplementary Table S1). Water suppression was incorporated into the second Z-filter, allowing to record spectra on hydrated samples.

**FIGURE 2:**
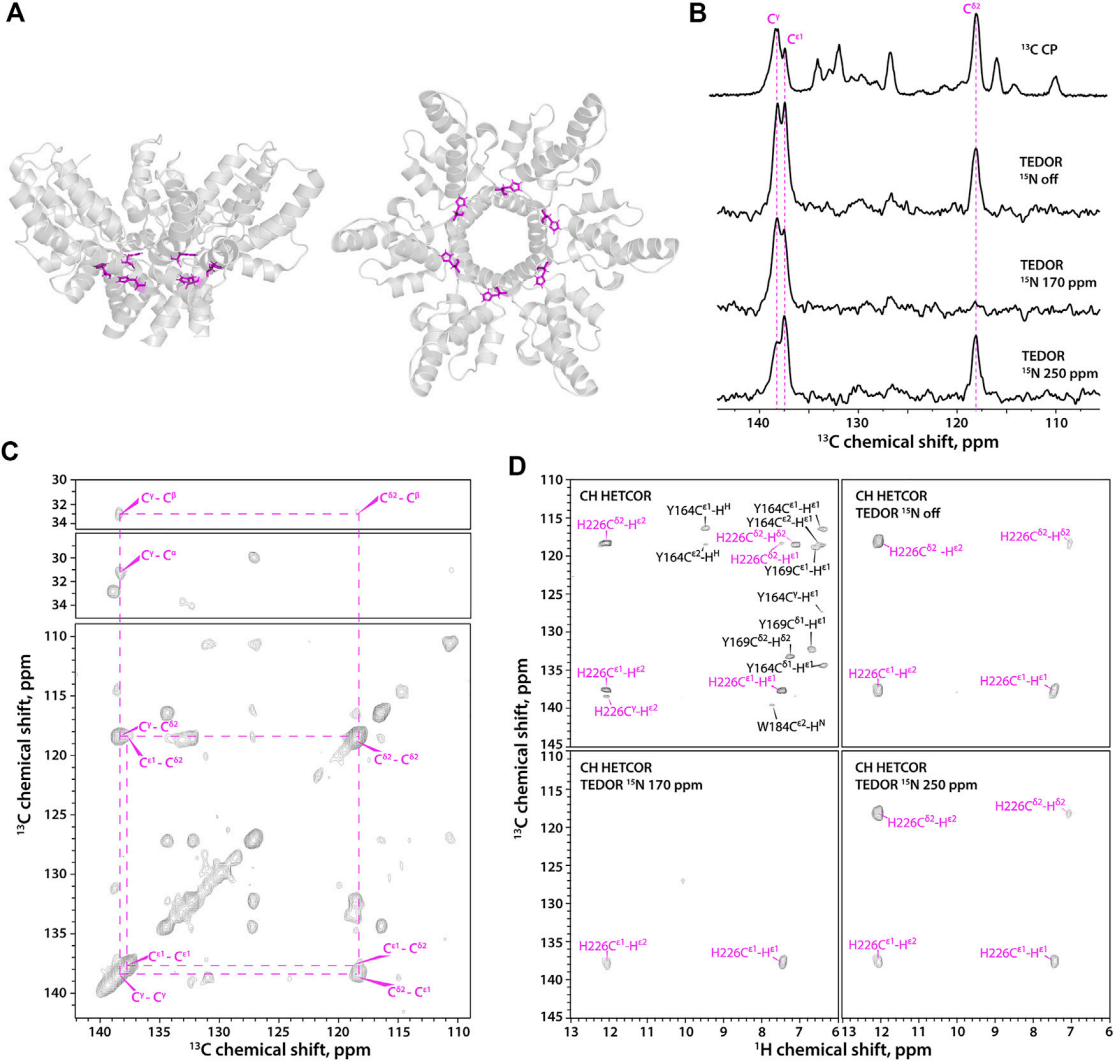
**(A)** A hexameric unit of HIV-1 CA_CTD_-SP1 in the microcrystalline assembly (PDB 5I4T) shown as side view **(left)** and top view **(right)**. **(B)** 1D ^13^C MAS NMR spectra of FD-^13^C,^15^N-CA_CTD_-SP1 with TEDOR-based ^15^N selective filtering in the aromatic region. Top to bottom: CPMAS spectrum; TEDOR-based ^15^N selectively filtered spectra with soft pulse turned off, soft pulse at 170 ppm, and soft pulse at 250 ppm. **(C)** 2D CORD spectrum of FD-^13^C,^15^N-CA_CTD_-SP1 (MAS frequency 14 kHz). **(D)** Aromatic regions of ^1^H-detected TEDOR-based ^15^N selective filtered CH HETCOR spectra in FD-^13^C,^15^N-CA_CTD_-SP1: TEDOR filter and soft pulse turned off **(top left)**, soft pulse turned off **(top right)**, soft pulse at 170 ppm **(bottom left)**, soft pulse at 250 ppm **(bottom right)**. The MAS frequency was 40 kHz in all experiments, unless indicated otherwise. Signals of τ tautomer are shown in magenta.

HIV-1 CA_CTD_-SP1 (Figure 2A) contains a single His residue, His-226. The outstanding high spectral resolution in the microcrystalline FD-^13^C,^15^N-CA_CTD_-SP1 sample allows for the determination of histidine protonation and tautomeric states even in the ^13^C-detected mode (Figure 2B). The C^ε1^ and C^γ^ resonances are present in 1D experiments, while the C^δ2^ resonance is absent in the ^15^N selective TEDOR-filtered ^13^C CPMAS experiment with the soft pulse at 170 ppm since its magnetization does not build up during the TEDOR block due to the very weak dipolar coupling to N^δ1^ (chemical shifts provided in Supplementary Table S2). The 2D ^13^C-^13^C CORD spectrum clearly shows a single set of resonances, indicating the presence of only one histidine species (Figure 2C), although the protonation and tautomeric state cannot be determined without additional experiments. The three complementary ^1^H-detected TEDOR-based ^15^N selective CH HETCOR spectra (Figure 2D) also indicate the presence of a single species, which is unambiguously assigned as τ tautomer. These ^1^H-detected 2D spectra contain no resonances of aromatic residues other than His (shown in black in the CH HETCOR spectrum) and Trp (these are weak or absent in the spectra of the deuterated sample), as only carbons attached to nitrogens are selected, making assignment of histidine resonances straightforward.

**FIGURE 3:**
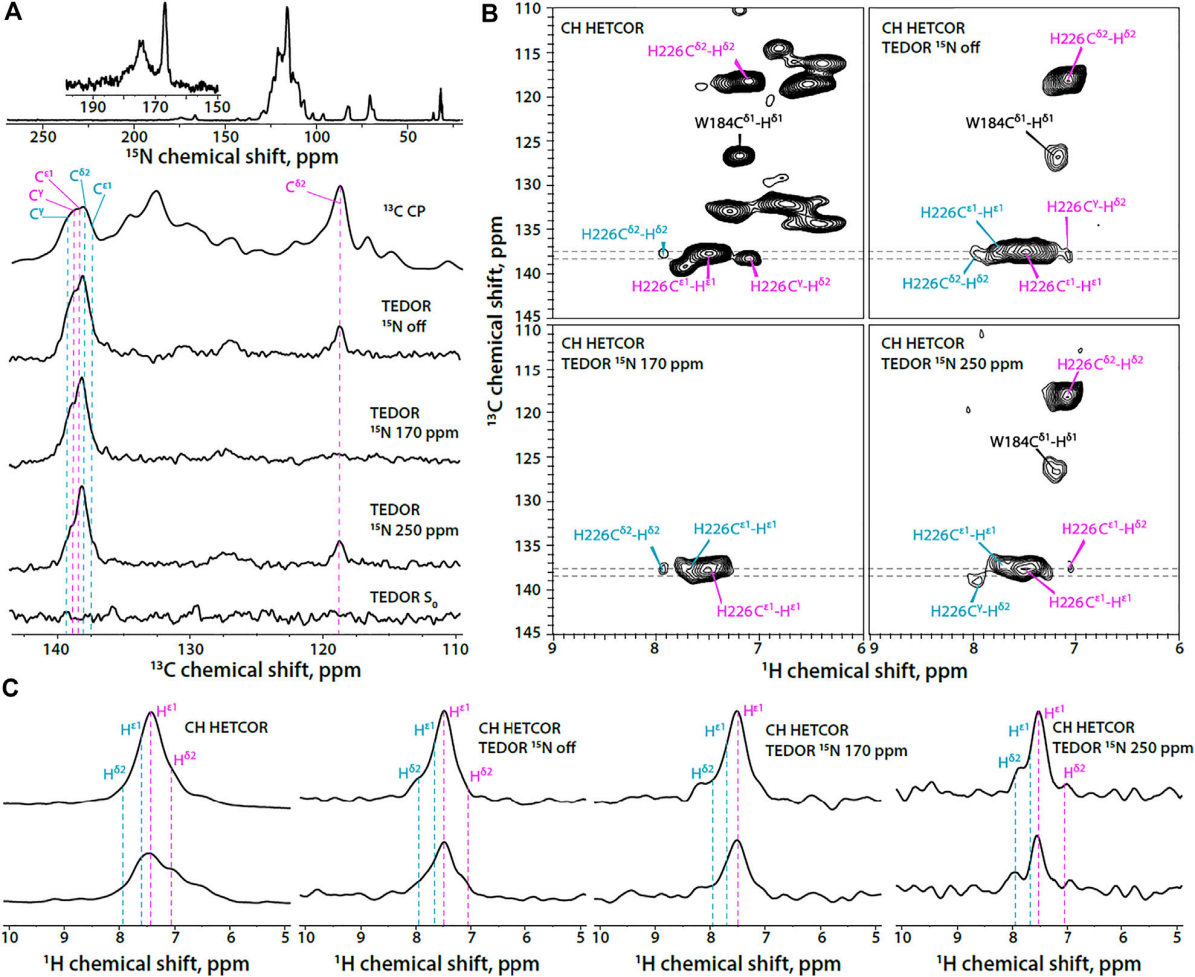
**(A)** 1D ^15^N CPMAS and ^13^C MAS NMR spectra of U-^13^C,^15^N-CA_CTD_-SP1 with TEDOR-based ^15^N selective filtering in the aromatic region (MAS frequency 14 kHz). Top to bottom: ^15^N CPMAS spectrum; ^13^C CPMAS spectrum; TEDOR-based ^15^N selectively filtered spectra with soft pulse turned off, soft pulse at 170 ppm, soft pulse at 250 ppm, and a reference (*S*_0_) experiment. **(B)** Aromatic regions of ^1^H-detected TEDOR-based ^15^N selective filtered CH HETCOR spectra in U-^13^C,^15^N-CA_CTD_-SP1: TEDOR filter and soft pulse turned off **(top left)**, soft pulse turned off **(top right)**, soft pulse at 170 ppm **(bottom left)**, soft pulse at 250 ppm **(bottom right)**. The first contour was set at 5× the noise rmsd. **(C)** 1D ^1^H slices of ^1^H-detected TEDOR-based ^15^N selective filtered CH HETCOR spectra in U-^13^C,^15^N-CA_CTD_-SP1, extracted at ^13^C shifts shown as gray dashed lines in panel **(B)**. Left to right: TEDOR filter and soft pulse turned off, soft pulse turned off, soft pulse at 170 ppm, soft pulse at 250 ppm. The MAS frequency was 60 kHz. Signals of τ tautomer and π tautomer are shown in magenta and teal, respectively.

In contrast to the FD-^13^C,^15^N-CA_CTD_-SP1, the His-226 protonation state in U-^13^C,^15^N-CA_CTD_-SP1 assemblies cannot be easily determined using the 1D ^13^C-detected version of TEDOR-based ^15^N selective filtered experiments due to low resolution and spectral overlap (Figure 3A). In contrast, the 2D ^1^H-detected TEDOR-based ^15^N selective filtered spectra (Figure 3B) suggest the presence of a small fraction of π tautomer along with the predominant τ tautomer in this sample (chemical shifts provided in Supplementary Table S2).

In addition to the His signals, the indole ring signals of the Trp184 residue are also present in the ^1^H-detected TEDOR-based experiments when the soft pulse is either turned off or centered at 250 ppm. This is expected due to the nitrogen atom N^ε1^ in the indole ring, which allows for magnetization build up on adjacent carbon atoms (C^δ1^ and C^ε2^) during TEDOR transfer. Tryptophan sidechain resonances appear much stronger in non-deuterated protein assemblies compared to the FD-^13^C,^15^N -CA_CTD_-SP1 and can be distinguished from those corresponding to the histidine based on chemical shift.

## Conclusion

We demonstrated that ^1^H-detected 2D Z-filtered TEDOR experiments incorporating ^15^N selective filters permit unambiguous assignment of histidine protonation and tautomeric states in microcrystalline proteins and protein assemblies. This approach combines all the advantages of fast MAS and proton detection. Extending the experiments to MAS frequencies of 110 kHz and above can further improve the quality of data sets and allow unambiguous assignment of His protonation and tautomeric states in larger proteins and protein assemblies.

## Data Availability

The raw data supporting the conclusions of this article will be made available by the authors, without undue reservation.

## References

[B1] Adams M. J. Blundell T. L. Dodson E. J. Dodson G. G. Vijayan M. Baker E. N. (1969). Structure of Rhombohedral 2 Zinc Insulin Crystals. Nature 224, 491–495. 10.1038/224491a0

[B2] Bachovchin W. W. Roberts J. D. (1978). Nitrogen-15 Nuclear Magnetic Resonance Spectroscopy. The State of Histidine in the Catalytic Triad of Alpha-Lytic Protease. Implications for the Charge-Relay Mechanism of Peptide-Bond Cleavage by Serine Proteases. J. Am. Chem. Soc. 100, 8041–8047. 10.1021/ja00494a001

[B3] Bertani P. Raya J. Bechinger B. (2014). ^15^N Chemical Shift Referencing in Solid State NMR. Solid State. Nucl. Magn. Reson. 61-62, 15–18. 10.1016/j.ssnmr.2014.03.003 24746715

[B4] Bloembergen N. (1949). On the Interaction of Nuclear Spins in a Crystalline Lattice. Physica 15, 386–426. 10.1016/0031-8914(49)90114-7

[B5] Blomberg F. Maurer W. Rueterjans H. (1977). Nuclear Magnetic Resonance Investigation of Nitrogen-15-Labeled Histidine in Aqueous Solution. J. Am. Chem. Soc. 99, 8149–8159. 10.1021/ja00467a005 925263

[B6] Blow D. M. Birktoft J. J. Hartley B. S. (1969). Role of a Buried Acid Group in the Mechanism of Action of Chymotrypsin. Nature 221, 337–340. 10.1038/221337a0 5764436

[B7] Campbell I. D. Lindskog S. White A. I. (1974). A Study of the Histidine Residues of Human Carbonic Anhydrase B Using 270 MHz Proton Magnetic Resonance. J. Mol. Biol. 90, 469–489. 10.1016/0022-2836(74)90229-0 4217387

[B8] Delaglio F. Grzesiek S. Vuister G. Zhu G. Pfeifer J. Bax A. (1995). NMRPipe: a Multidimensional Spectral Processing System Based on UNIX Pipes. J. Biomol. NMR 6, 277–293. 10.1007/bf00197809 8520220

[B9] Edwards B. F. P. Sykes B. D. (1980). Nuclear Magnetic Resonance Evidence for the Coexistence of Several Conformational States of Rabbit Cardiac and Skeletal Tropomyosins. Biochemistry 19, 2577–2583. 10.1021/bi00553a007 7397090

[B10] French T. C. Hammes G. G. (1965). Relaxation Spectra of Ribonuclease. II. Isomerization of Ribonuclease at Neutral pH Values. J. Am. Chem. Soc. 87, 4669–4673. 10.1021/ja00949a002 5844452

[B11] Goddard T. D. Kneller D. G. (2004). SPARKY 3. Univ. California, San Fransisco.

[B12] Hansen A. L. Kay L. E. (2014). Measurement of Histidine pKa Values and Tautomer Populations in Invisible Protein States. Proc. Natl. Acad. Sci. 111, E1705–E1712. 10.1073/pnas.1400577111 24733918 PMC4035968

[B13] Hass M. A. S. Hansen D. F. Christensen H. E. M. Led J. J. Kay L. E. (2008). Characterization of Conformational Exchange of a Histidine Side Chain: Protonation, Rotamerization, and Tautomerization of His61 in Plastocyanin from Anabaena Variabilis. J. Am. Chem. Soc. 130, 8460–8470. 10.1021/ja801330h 18540585

[B14] Hoffee P. Lai C. Y. Pugh E. L. Horecker B. L. (1967). The Function of Histidine Residues in Rabbit Muscle Aldolase. Proc. Natl. Acad. Sci. 57, 107–113. 10.1073/pnas.57.1.107 5233166 PMC335471

[B15] Hou G. Yan S. Trébosc J. Amoureux J.-P. Polenova T. (2013). Broadband Homonuclear Correlation Spectroscopy Driven by Combined R2_n_ ^v^ Sequences Under Fast Magic Angle Spinning for NMR Structural Analysis of Organic and Biological Solids. J. Magn. Reson. 232, 18–30. 10.1016/j.jmr.2013.04.009 23685715 PMC3703537

[B16] Hu J. Fu R. Nishimura K. Zhang L. Zhou H.-X. Busath D. D. (2006). Histidines, Heart of the Hydrogen Ion Channel from Influenza A Virus: toward an Understanding of Conductance and Proton Selectivity. Proc. Natl. Acad. Sci. 103, 6865–6870. 10.1073/pnas.0601944103 16632600 PMC1458985

[B17] Hu F. Luo W. Hong M. (2010). Mechanisms of Proton Conduction and Gating in Influenza M2 Proton Channels from Solid-State NMR. Science 330, 505–508. 10.1126/science.1191714 20966251 PMC4102303

[B18] Kilmartin J. V. Breen J. J. Roberts G. C. K. Ho C. (1973). Direct Measurement of the pK Values of an Alkaline Bohr Group in Human Hemoglobin. Proc. Natl. Acad. Sci. 70, 1246–1249. 10.1073/pnas.70.4.1246 4515623 PMC433468

[B19] Kossiakoff A. A. Spencer S. A. (1980). Neutron Diffraction Identifies His 57 as the Catalytic Base in Trypsin. Nature 288, 414–416. 10.1038/288414a0 7432541

[B20] Kossiakoff A. A. Spencer S. A. (1981). Direct Determination of the Protonation States of Aspartic Acid-102 and Histidine-57 in the Tetrahedral Intermediate of the Serine Proteases: Neutron Structure of Trypsin. Biochemistry 20, 6462–6474. 10.1021/bi00525a027 7030393

[B21] Kovalevsky A. Y. Chatake T. Shibayama N. Park S.-Y. Ishikawa T. Mustyakimov M. (2010). Direct Determination of Protonation States of Histidine Residues in a 2 Å Neutron Structure of Deoxy-Human Normal Adult Hemoglobin and Implications for the Bohr Effect. J. Mol. Biol. 398, 276–291. 10.1016/j.jmb.2010.03.016 20230836

[B22] Kupce E. Boyd J. Campbell I. D. (1995). Short Selective Pulses for Biochemical Applications. J. Magn. Reson. Ser. B 106, 300–303. 10.1006/jmrb.1995.1049 7719630

[B23] Kwon B. Roos M. Mandala V. S. Shcherbakov A. A. Hong M. (2019). Elucidating Relayed Proton Transfer through a His-Trp-His Triad of a Transmembrane Proton Channel by Solid-State NMR. J. Mol. Biol. 431, 2554–2566. 10.1016/j.jmb.2019.05.009 31082440 PMC6589385

[B24] Lee W. Tonelli M. Markley J. L. (2015). NMRFAM-SPARKY: Enhanced Software for Biomolecular NMR Spectroscopy. Bioinformatics 31, 1325–1327. 10.1093/bioinformatics/btu830 25505092 PMC4393527

[B25] Lewis S. D. Johnson F. A. Shafer J. A. (1976). Potentiometric Determination of Ionizations at the Active Site of Papain. Biochemistry 15, 5009–5017. 10.1021/bi00668a010 10964

[B26] Lewis S. D. Johnson F. A. Shafer J. A. (1981). Effect of Cysteine-25 on the Ionization of Histidine-159 in Papain as Determined by Proton Nuclear Magnetic Resonance Spectroscopy. Evidence for a Histidine-159-Cysteine-25 Ion Pair and its Possible Role in Catalysis. Biochemistry 20, 48–51. 10.1021/bi00504a009 7470479

[B27] Li S. Hong M. (2011). Protonation, Tautomerization, and Rotameric Structure of Histidine: a Comprehensive Study by Magic-Angle-Spinning Solid-State NMR. J. Am. Chem. Soc. 133, 1534–1544. 10.1021/ja108943n 21207964 PMC4082993

[B28] Liljas A. Kannan K. K. Bergstén P.-C. Waara I. Fridborg K. Strandberg B. (1972). Crystal Structure of Human Carbonic Anhydrase C. Nat. New Biol. 235, 131–137. 10.1038/newbio235131a0 4621826

[B29] Loewenthal R. Sancho J. Fersht A. R. (1992). Histidine-aromatic Interactions in Barnase. J. Mol. Biol. 224, 759–770. 10.1016/0022-2836(92)90560-7 1569555

[B30] Maciejko J. Kaur J. Becker-Baldus J. Glaubitz C. (2019). Photocycle-dependent Conformational Changes in the Proteorhodopsin Cross-Protomer Asp-His-Trp Triad Revealed by DNP-Enhanced MAS-NMR. Proc. Natl. Acad. Sci. USA 116, 8342–8349. 10.1073/pnas.1817665116 30948633 PMC6486740

[B31] Maeda M. Chatake T. Tanaka I. Ostermann A. Niimura N. (2004). Crystallization of a Large Single crystal of Cubic Insulin for Neutron Protein Crystallography. J. Synchrotron Radiat. 11, 41–44. 10.1107/s0909049503023859 14646130

[B32] Mance D. Sinnige T. Kaplan M. Narasimhan S. Daniëls M. Houben K. (2015). An Efficient Labelling Approach to Harness Backbone and Side‐Chain Protons in ^1^H‐Detected Solid‐State NMR Spectroscopy. Angew. Chem. Int. Ed. 54, 15799–15803. 10.1002/anie.201509170 PMC4691318 26555653

[B33] Markley J. L. (1975). Observation of Histidine Residues in Proteins by Nuclear Magnetic Resonance Spectroscopy. Acc. Chem. Res. 8, 70–80. 10.1021/ar50086a004

[B34] Miao Y. Cross T. A. Fu R. (2014). Differentiation of Histidine Tautomeric States Using ^15^N Selectively Filtered ^13^C Solid-State NMR Spectroscopy. J. Magn. Reson. 245, 105–109. 10.1016/j.jmr.2014.06.005 25026459 PMC4136442

[B35] Miao Y. Fu R. Zhou H.-X. Cross T. A. (2015). Dynamic Short Hydrogen Bonds in Histidine Tetrad of Full-Length M2 Proton Channel Reveal Tetrameric Structural Heterogeneity and Functional Mechanism. Structure 23, 2300–2308. 10.1016/j.str.2015.09.011 26526851 PMC4670578

[B36] Morcombe C. R. Zilm K. W. (2003). Chemical Shift Referencing in MAS Solid State NMR. J. Magn. Reson. 162, 479–486. 10.1016/S1090-7807(03)00082-X 12810033

[B37] Movellan K. T. Wegstroth M. Overkamp K. Leonov A. Becker S. Andreas L. B. (2020). Imidazole-Imidazole Hydrogen Bonding in the pH-Sensing Histidine Side Chains of Influenza A M2. J. Am. Chem. Soc. 142, 2704–2708. 10.1021/jacs.9b10984 31970979 PMC7307898

[B38] Munowitz M. Bachovchin W. W. Herzfeld J. Dobson C. M. Griffin R. G. (1982). Acid-base and Tautomeric Equilibriums in the Solid State: Nitrogen-15 NMR Spectroscopy of Histidine and Imidazole. J. Am. Chem. Soc. 104, 1192–1196. 10.1021/ja00369a007

[B39] Pelton J. G. Torchia D. A. Meadow N. D. Roseman S. (1993). Tautomeric States of the Active-Site Histidines of Phosphorylated and Unphosphorylated IIIGlc, a Signal-Transducing Protein from *Escherichia coli*, Using Two-Dimensional Heteronuclear NMR Techniques. Protein Sci. 2, 543–558. 10.1002/pro.5560020406 8518729 PMC2142369

[B40] Perutz M. F. Mathews F. S. (1966). An X-ray Study of Azide Methaemoglobin. J. Mol. Biol. 21, 199–202. 10.1016/0022-2836(66)90088-x 5969763

[B41] Perutz M. F. Muirhead H. Mazzarella L. Crowther R. A. Greer J. Kilmartin J. V. (1969). Identification of Residues Responsible for the Alkaline Bohr Effect in Haemoglobin. Nature 222, 1240–1243. 10.1038/2221240a0 5789657

[B42] Perutz M. F. Gronenborn A. M. Clore G. M. Fogg J. H. Shih D. T.-b. (1985). The pKa Values of Two Histidine Residues in Human Haemoglobin, the Bohr Effect, and the Dipole Moments of α-helices. J. Mol. Biol. 183, 491–498. 10.1016/0022-2836(85)90016-6 4020866

[B43] Platzer G. Okon M. Mcintosh L. P. (2014). pH-Dependent Random Coil ^1^H, ^13^C, and ^15^N Chemical Shifts of the Ionizable Amino Acids: a Guide for Protein pK a Measurements. J. Biomol. NMR 60, 109–129. 10.1007/s10858-014-9862-y 25239571

[B44] Plesniak L. A. Connelly G. P. Mcintosh L. P. Wakarchuk W. W. (1996). Characterization of a Buried Neutral Histidine Residue in Bacillus Circulansxylanase: NMR Assignments, pH Titration, and Hydrogen Exchange. Protein Sci. 5, 2319–2328. 10.1002/pro.5560051118 8931150 PMC2143293

[B45] Shenderovich I. G. Lesnichin S. B. Tu C. Silverman D. N. Tolstoy P. M. Denisov G. S. (2015). NMR Studies of Active-Site Properties of Human Carbonic Anhydrase II by Using ^15^N-Labeled 4-Methylimidazole as a Local Probe and Histidine Hydrogen-Bond Correlations. Chem. Eur. J. 21, 2915–2929. 10.1002/chem.201404083 25521423

[B46] Shimba N. Takahashi H. Sakakura M. Fujii I. Shimada I. (1998). Determination of Protonation and Deprotonation Forms and Tautomeric States of Histidine Residues in Large Proteins Using Nitrogen−Carbon J Couplings in Imidazole Ring. J. Am. Chem. Soc. 120, 10988–10989. 10.1021/ja982153g

[B47] Stevens T. J. Fogh R. H. Boucher W. Higman V. A. Eisenmenger F. Bardiaux B. (2011). A Software Framework for Analysing Solid-State MAS NMR Data. J. Biomol. NMR 51, 437–447. 10.1007/s10858-011-9569-2 21953355 PMC3222832

[B48] Stryer L. Kendrew J. C. Watson H. C. (1964). The Mode of Attachment of the Azide Ion to Sperm Whale Metmyoglobin. J. Mol. Biol. 8, 96–IN10. 10.1016/s0022-2836(64)80152-2 14149967

[B49] Vasa S. K. Singh H. Grohe K. Linser R. (2019). Assessment of a Large Enzyme-Drug Complex by Proton‐Detected Solid‐State NMR Spectroscopy without Deuteration. Angew. Chem. Int. Ed. 58, 5758–5762. 10.1002/anie.201811714 30688395

[B50] Vinod Chandran C. Madhu P. K. Kurur N. D. Bräuniger T. (2008). Swept-frequency Two-Pulse Phase Modulation (SWf-TPPM) Sequences with Linear Sweep Profile for Heteronuclear Decoupling in Solid-State NMR. Magn. Reson. Chem. 46, 943–947. 10.1002/mrc.2285 18666219

[B51] Wagner J. M. Zadrozny K. K. Chrustowicz J. Purdy M. D. Yeager M. Ganser-Pornillos B. K. (2016). Crystal Structure of an HIV Assembly and Maturation Switch. eLife 5, e17063. 10.7554/eLife.17063 27416583 PMC4946879

[B52] Wei Y. De Dios A. C. McDermott A. E. (1999). Solid-State ^15^N NMR Chemical Shift Anisotropy of Histidines: Experimental and Theoretical Studies of Hydrogen Bonding. J. Am. Chem. Soc. 121, 10389–10394. 10.1021/ja9919074

[B53] Zhou D. H. Rienstra C. M. (2008). High-performance Solvent Suppression for Proton Detected Solid-State NMR. J. Magn. Reson. 192, 167–172. 10.1016/j.jmr.2008.01.012 18276175 PMC2443633

[B54] Zhou M. M. Davis J. P. Van Etten R. L. (1993). Identification and pKa Determination of the Histidine Residues of Human Low-Molecular-Weight Phosphotyrosyl Protein Phosphatases: A Convenient Approach Using MLEV-17 Spectral Editing Scheme. Biochemistry 32, 8479–8486. 10.1021/bi00084a012 7689332

