# Supplementary material for: Determination of Histidine Protonation States in Proteins by Fast Magic Angle Spinning NMR
Source: Front Mol Biosci. 2021 Dec 10;8:767040. doi: 10.3389/fmolb.2021.767040 (PMC8703106; doi:10.3389/fmolb.2021.767040)
Supplement: Supplementary file 1 [file DataSheet1.PDF]

Supplementary Material

Table S1. ^{1}H, ^{13}C and ^{15}N isotropic chemical shifts* (ppm) of Histidine in different protonation and tautomeric state.

site		Li and Hong, 2011			This work	
		cationic	τ tautomer	π tautomer	cationic	τ tautomer
^{13}C	C'	173.2	175.6	183.2	174.6	177.3
	Cα	54.1	57.0	59.4	55.7	58.8
	Cβ	26.0	27.0	28.4	28.5	28.5
	Cγ	128.7	137.7	129.2	130.0	139.3
	Cε1	136.3	135.3	135.8	137.8	137.2
	Cδ2	119.4	113.6	125.4	120.8	115.4
^{15}N	Nα	47.6	41.5	96.3	48.1	42.0
	Nδ1	190.0	249.4	171.8	190.2	249.9
	Nε2	176.3	171.1	248.2	176.9	171.7
^{1}H	HN	8.6	9.0	6.2	8.0	8.5
	Hα	3.5	4.3	4.0	3.3	4.5
	Hβ	3.3	2.7	2.7	2.2	2.2
	Hδ1	16.8	NA	12.7	16.7	NA
	Hε2	12.6	13.7	NA	12.1	13.3
	Hδ2	8.0	4.9	6.4	7.1	4.2
	Hε1	9.3	6.1	7.2	8.6	5.3

* ^{13}C chemical shifts in Li and Hong, 2011 are referenced to TMS, while in this work chemical shifts are referenced to DSS giving rise to a 2.5 ppm difference between reported values

Table S2. ^{1}H, ^{13}C and ^{15}N isotropic chemical shifts* (ppm) of Histidine 226 in FD-^{13}C,^{15}N-CA$_{CTD}$-SP1 and U-^{13}C,^{15}N-CA$_{CTD}$-SP1 reported in this work.

Sample	Residue	site		δ_{iso}, ppm
FD-^{13}C,^{15}N-CA$_{CTD}$-SP1	H226(τ)	^{13}C	Cγ	138.4
			Cε1	137.6
			Cδ2	118.3
		^{1}H	Hε2	12.1
			Hδ2	7.1
			Hε1	7.45
U-^{13}C,^{15}N-CA$_{CTD}$-SP1	H226(τ)	^{13}C	Cγ	138.4
			Cε1	137.8
			Cδ2	118.4
		^{1}H	Hε2	-
			Hδ2	7.1
			Hε1	7.5
		^{15}N	Nε2	168.2
	H226(π)	^{13}C	Cγ	139.5
			Cε1	137.2
			Cδ2	137.7
		^{1}H	Hδ2	-
			Hδ2	7.95
			Hε1	7.7
		^{15}N	Nδ1	176

References

Li, S., and Hong, M. (2011). Protonation, tautomerization, and rotameric structure of histidine: a comprehensive study by magic-angle-spinning solid-state NMR. *J. Am. Chem. Soc.* 133**,** 1534-1544. doi:10.1021/ja108943n
